# Intraoperative detection of stuck leaflet after implantation of a mechanical aortic valve: a case report

**DOI:** 10.1186/s40981-020-0313-3

**Published:** 2020-02-07

**Authors:** Kazuto Miyata, Sayaka Shigematsu

**Affiliations:** Department of Anesthesia, New Heart Watanabe Institute, Hamadayama 3-19-11, Suginami-ku, Tokyo, 168-0065 Japan

**Keywords:** Stuck leaflet, Mechanical aortic valve, Suture materials

## Abstract

**Background:**

Mechanical aortic valve dysfunction is a rare but potentially fatal complication. It is usually caused by thrombosis, vegetation, and pannus overgrowth. However, it very rarely occurs immediately after the cardiopulmonary bypass weaning period.

**Case presentation:**

We describe a case of stuck leaflet after the implantation of a mechanical aortic valve because of residual suture materials interfering with mechanical aortic valve leaflet closure, which is a very rare cause and has not been reported until now.

**Conclusion:**

The residual suture materials for mechanical aortic valve replacement could cause mechanical valve dysfuction indicated as stuck leafet.

## Background

Evaluation using intraoperative transesophageal echocardiography (IO-TEE) of a mechanical heart valve immediately after cardiopulmonary bypass (CPB) weaning is needed to make a decision on whether repeat CPB is required. Mechanical aortic valve dysfunction is a very rare complication and is usually due to thrombosis, vegetation, and pannus overgrowth, which cannot occur immediately after CPB. IO-TEE shows that normal transvalvular leaks occur from hinge points, and the degree of leakage is usually trivial and symmetric in mechanical prosthetic valves.

Here, we report a very rare case of stuck leaflet of prosthetic aortic valve, and IO-TEE show that abnormal transvalvular leakage caused by residual suture materials interfering with leaflet closure, which has not been reported until now. Written consent was obtained from the patient to publish this case report.

## Case presentation

A 57-year-old woman (155 cm; 65 kg) with aortic valve stenosis (aortic valve area, 0.77 cm^2^) and left ventricular pressure overload hypertrophy with a normal systolic function was scheduled to undergo mechanical aortic valve replacement. She had a history of hypertension, hyperlipidemia, and type 2 diabetes mellitus, for which she was being treated with an angiotensin receptor antagonist, statin, and insulin, respectively.

General anesthesia was induced with 4 mg of midazolam, 50 mg of rocuronium, and 0.3 μg/kg/min of remifentanil intravenously. After intubation, an IO-TEE probe was inserted. A central venous catheter and pulmonary artery catheter were placed in the right internal jugular vein. Anesthesia was maintained with sevoflurane 1.5% in oxygen and air and continuous infusions of remifentanil 0.2–0.4 μg/kg/min and propofol 4 mg/kg/h.

IO-TEE showed a thickened tricuspid aortic valve with an immobile cusp and mild aortic regurgitation, with no other valve insufficiency. The aortic annulus diameter was 19 mm.

**Fig. 1:**
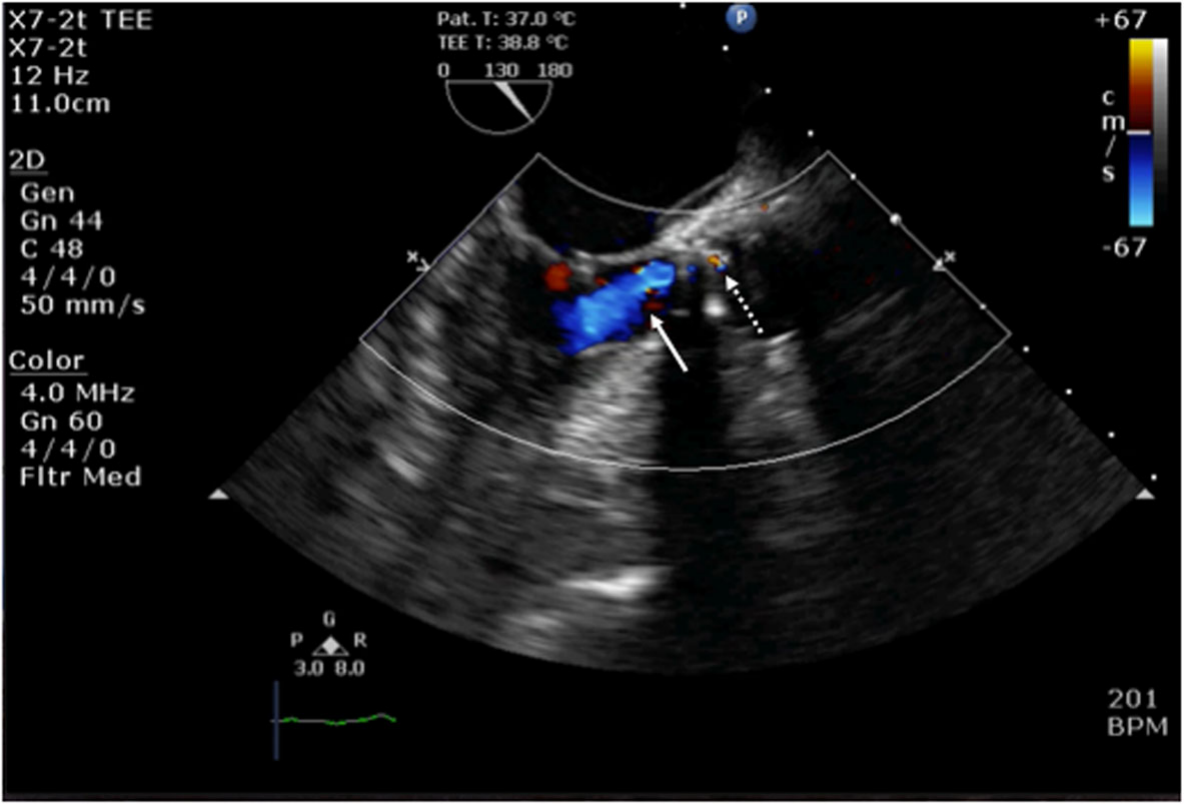
A mid-esophageal aortic valve long-axis transesophageal echocardiography image reveals an aortic regurgitation jet and its acceleration flow. White arrow indicates aortic regurgitation, and white dotted arrow indicates its acceleration flow

CPB was performed in a standard manner. After aortic cross-clamping, the native aortic valve was resected and replaced with an 18-mm mechanical aortic valve (ATS-AP360, Medtonic Inc., USA) using the non-evert mattress suture technique. After rewarming, the heart was de-aired and the cross-clamp was removed. After declamping of the aorta, a 3-μg/kg/min dopamine infusion was started. IO-TEE was performed to study the new prosthetic aortic valve before weaning off CPB (Fig. 1).

**Fig. 2:**
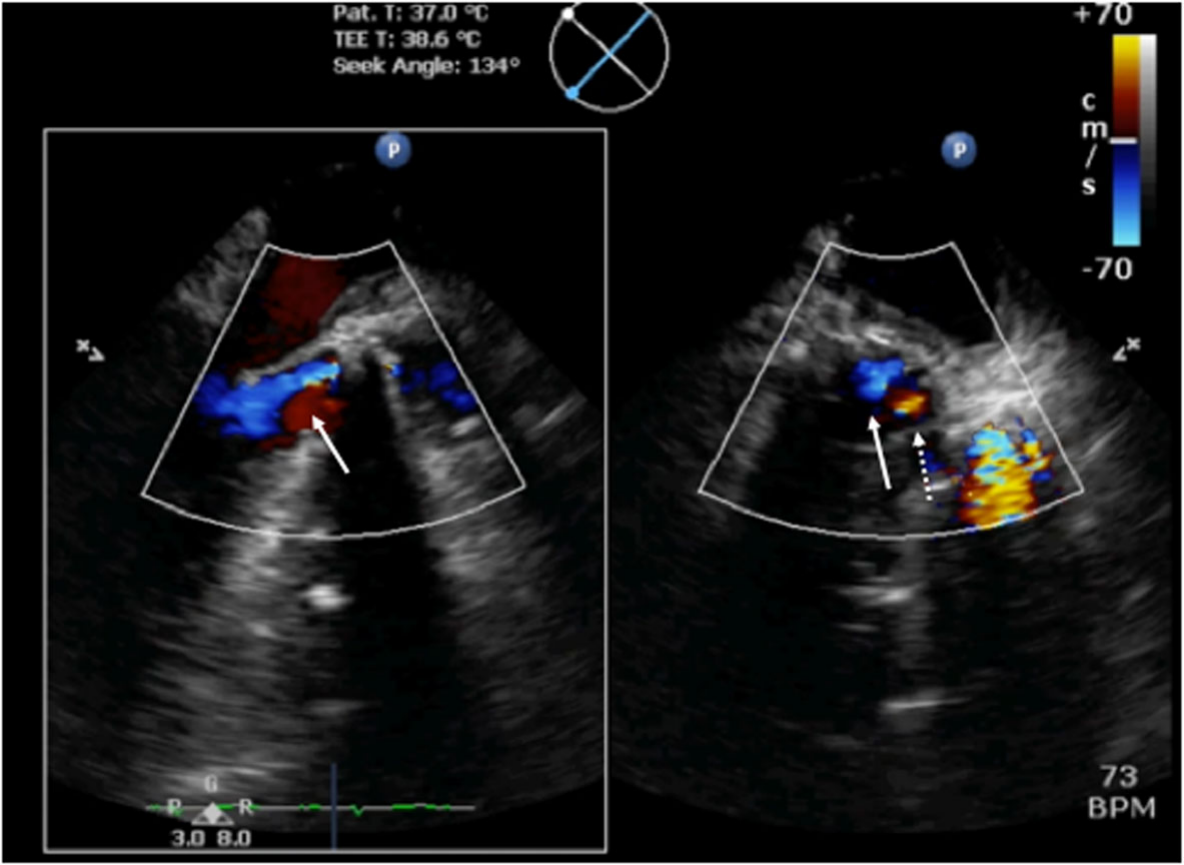
Using LIVE xPlane, a mid-esophageal aortic valve long-axis transesophageal echocardiography image (left image) and aortic valve short-axis transesophageal echocardiography image (right image) were taken, with the image inverted from right to left. Acceleration flow was recognized in the non-coronary cusp of the intra-sewing ring white arrow indicates aortic regurgitation and white dotted arrow indicates its acceleration flow

The mid-esophageal aortic valve short-axis view revealed that the mechanical bileaflet prosthetic valves were inserted in an ordinary position, which indicated that the two hinges were not located on the coronary ostia. The mid-esophageal aortic valve long-axis view revealed the presence of aortic regurgitation jets (Fig. 1), toward the anterolateral papillary muscle. The width of the regurgitation jet was 0.73 cm, indicating severe aortic regurgitation. LIVE xPlane, using a CX50 (Philips Medical Systems, Bothell, WA, USA), revealed that the regurgitation had originated in the intra-sewing ring of a non-coronary cusp and was not located at the point of the hinges (Fig. 2). At the time, the cause of the abnormal severe transvalvular leakage was unknown. Thus, we decided to perform a second CPB. After re-aortic-cross-clamping and aortotomy, residual suture material placed in the sewing ring was observed to be caught in one leaflet, interfering with the closing of the leaflet. Upon cutting the residual suture material, the leaflet began to move appropriately, and non-interference with the leaflet motion was confirmed. Weaning from the second CPB was very smooth. After CPB weaning, IO-TEE showed no transvalvular leakage into the mechanical prosthetic valve. The postoperative course was uneventful.

## Discussion

We found out two important clinical issues in this case report. Residual suture materials using valve replacement could interfer with leaflet motion in aoritic valve position. It is difficult to diagnose stuck leaflet in the aortic valve position because of an acoustic shadow. It is useful to distinguish between transvalvular and paravalvular leakage of abnormal regurgitation to diagnose stuck leaflet, and it is essential to use multiple visualization views, such as those provided by LIVE xPlane.

The most common cause of mechanical aortic valve dysfunction is pannus formation [1–3]. Other causes include entrapment of the leaflet by thrombus, vegetation, and subvalvular tissue. Pannus, thrombus, and vegetation are unlikely to arise immediately after implantation of the aortic valve. Further, in the aortic position, the subvalvular tissues consist of the sinus of Valsalva, which is unlikely to interfere with disk motion. In the present case, an intermittent stuck leaflet was considered to be caused by residual suture materials, which has not been reported previously. The vast majority of reports of the stuck valve immediately after CPB were in the mitral position. There have never been in the aortic position. Table 1 summarizes the case reports of prosthesis valve dysfunction immediately after CPB [4–6].

**Table 1:** Reports of stuck mitral valves immediately after cardiopulmonary bypass

Author	Mechanisms	Type of valves	Management
Fujii H, et al [4]	unclear	mechanical	90° rotation
Fujii H, et al [4]	suture loop gaming	bioprosthesis	replacement
Murugesan C, et al [5]	tertiary chord between disc and ring	mechanical	removal of chord element
Raut MS, et al [6]	unclear	mechanical	90° rotation

Abnormal regurgitation can be divided into transvalvular and/or paravalvular regurgitation. In general, mechanical bileaflet prosthetic valves have two hinges that allow pivot motion. Normal transvalvular leaks involve four trivial jets at the four hinge points, preventing blood stasis. Evaluation of the origin of the acceleration flow is very important in distinguishing between transvalvular and paravalvular leaks. However, because of acoustic shadowing, they can be difficult to visualize, especially in the case of mechanical bileaflet prosthetic valves. Thus, it is essential to use multiple visualization views, such as those provided by LIVE xPlane. If paravalvular leakage occurs, acceleration flow is found in the extra-sewing ring. However, in this case, as the acceleration flow originated in the intra-sewing ring, we diagnosed transvalvular leakage. And in the short-axis view, the acceleration flow was shown red and transvalvular leakage was shown blue, because these flows are approached or left to ultrasonic probe. However, the cause of transvalvular leakage could not be diagnosed by only IO-TEE. After re-aortic-cross-clamping and aortotomy, we finally diagnose that residual suture materials interfering with mechanical aortic valve leaflet closure.

## Conclusion

Immediately after the CPB, mechanical aortic valve dysfunction seldom occurred. The present case is a very rare case of transvalvular leakage of a bileaflet mechanical prosthetic valve caused by residual suture materials that interfered with the closing of the leaflet.

## Data Availability

Not applicable

## Abbreviations

CPB: Cardiopulmonary bypass
TEE: Transesophageal echocardiography
